# Disruption of *AP3B1* by a chromosome 5 inversion: a new disease mechanism in Hermansky-Pudlak syndrome type 2

**DOI:** 10.1186/1471-2350-14-42

**Published:** 2013-04-04

**Authors:** Matthew L Jones, Sherina L Murden, Claire Brooks, Viv Maloney, Richard A Manning, Kimberly C Gilmour, Vandana Bharadwaj, Josu de la Fuente, Subarna Chakravorty, Andrew D Mumford

**Affiliations:** 1 Bristol Heart Institute & School of Cellular and Molecular Medicine, University of Bristol, Bristol, UK; 2 North West Thames Regional Genetics Service, Northwick Park Hospital, Harrow, UK; 3 Wessex Regional Genetics Laboratory, Salisbury District Hospital, Salisbury, UK; 4 Department of Haematology, Imperial College Academic Health Care Trust, Hammersmith Hospital, London, UK; 5 Department of Immunology, Great Ormond Street Hospital for Children NHS Trust, London, UK; 6 Department of Medicine, Imperial College, London, UK; 7 Department of Paediatric Haematology, St Marys Hospital, Imperial College Healthcare NHS Trust, London, UK

**Keywords:** Chromosome 5 inversion, Hermansky-Pudlak syndrome type 2, Adaptor-related protein complex 3 β3A subunit, Fluorescence *in situ* hybridisation

## Abstract

**Background:**

Hermansky-Pudlak syndrome 2 (HPS2; OMIM #608233) is a rare, autosomal recessive disorder caused by loss-of-function genetic variations affecting *AP3B1*, which encodes the β3A subunit of the adaptor-related protein complex 3 (AP3). Phenotypic characteristics include reduced pigmentation, absent platelet dense granule secretion, neutropenia and reduced cytotoxic T lymphocyte (CTL) and natural killer (NK) cell function. To date HPS2 has been associated with non-synonymous, stop-gain or deletion-insertion nucleotide variations within the coding region of *AP3B1*.

**Case presentation:**

We describe a consanguineous female infant with reduced pigmentation, neutropenia and recurrent infections. Platelets displayed reduced aggregation and absent ATP secretion in response to collagen and ADP, indicating a platelet dense granule defect. There was increased basal surface expression of CD107a (lysosome-associated membrane protein 1(LAMP-1)) on NK cells and CTLs from the study subject and a smaller increase in the percentage of CD107a positive cells after stimulation compared to most healthy controls. Immunoblotting of protein extracts from EBV-transformed lymphoblasts from the index case showed absent expression of full-length AP-3 β3A subunit protein, confirming a phenotypic diagnosis of HPS2.

The index case displayed a homozygous pericentric inv(5)(p15.1q14.1), which was also detected as a heterozygous defect in both parents of the index case. No loss of genetic material was demonstrated by microarray comparative genome hybridisation at 60kb resolution. Fluorescence *in-situ* hybridisation using the 189.6kb probe RP11-422I12, which maps to 5q14.1, demonstrated dual hybridisation to both 5q14.1 and 5p15.1 regions of the inverted Chr5. The RP11-422I12 probe maps from intron 1 to intron 16 of *AP3B1,* thus localising the 5q inversion breakpoint to within *AP3B1.* The probe RP11-211K15, which corresponds to an intergenic region on 5p also showed dual hybridisation, enabling localisation of the 5p inversion breakpoint.

**Conclusion:**

This case report extends the phenotypic description of the very rare disorder HPS2. Our demonstration of a homozygous Chr5 inversion predicted to disrupt *AP3B1* gene provides a novel pathogenic mechanism for this disorder.

## Background

Hermansky-Pudlak syndrome (HPS) is a group of genetically heterogeneous disorders that are caused by mutations in genes that affect the synthesis and function of lysosome-related organelles (LRO) [1,2]. LRO are specialised structures that have the ultra-structural and functional characteristics of lysosomes but serve as secretory granules in some cell types. They are widely distributed in haematopoietic and other tissues and include melanosomes in melanocytes, dense granules in platelets, lamellar bodies in alveolar type II epithelial cells, azurophilic granules in neutrophils and lytic granules in cytotoxic T lymphocytes (CTL) and natural killer (NK) cells [3]. In keeping with the distribution of LRO, abnormal LRO synthesis or function in HPS manifests as a complex multi-organ phenotype. Features common to all HPS sub-types include reduced pigmentation caused by absence of melanosomes and a mild bleeding diathesis caused by absence of platelet dense granules. Other phenotype features differ according to HPS subtype but may include respiratory fibrosis, colitis and immunodeficiency [1,2].

HPS type 2 (HPS2) is a very rare subtype of HPS in which reduced pigmentation and platelet dysfunction are accompanied by neutropenia [4-13] and in some cases, reduced CTL and NK cell cytotoxicity. This may manifest as increased susceptibility to viral infection and rarely, haemophagocytic lymphohistiocytosis (HLH) in response to viral infection [7,8,12]. HPS2 has previously been associated with loss-of-function nucleotide variations in *AP3B1* that encodes the β3A-subunit of the adaptor protein complex 3 (AP-3) [11]. AP-3 is essential for trafficking cargo proteins to LRO. Consequently, proteins such as CD63 and LAMP-1, which are normally targeted to the lysosome compartment in haematopoietic cells, are incorrectly trafficked to the cell surface and accumulate in endosomes [4]. In all the previous reports of HPS2, loss of AP-3 β3A-subunit expression is associated with missense, nonsense or deletion-insertion nucleotide variations within *AP3B1*. Here we report the clinical and laboratory phenotype of a new index case with HPS2 associated with a homozygous pericentric inversion of Chr5. We demonstrate that the 5q inversion breakpoint lies within *AP3B1* thus providing a new mechanistic explanation for the HPS2 phenotype.

## Case presentation

**Figure 1:**
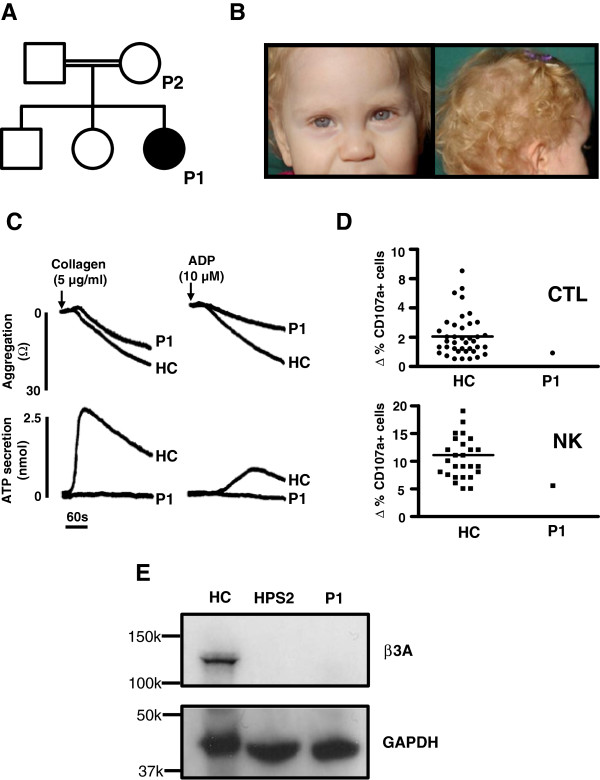
**Clinical and laboratory phenotype of the index case.** Pedigree (**A**) showing the index case (P1; shaded) who displayed reduced skin, hair and iris pigmentation (**B**). Whole blood lumiaggregometry (**C**) was performed on EDTA-anticoagulated blood from P1 and from a healthy control (HC) using the activating agonists collagen (5 μg/ml) and ADP (10 μM). In the top panel, platelet aggregation is indicated by change in electrical impedance after addition of the agonist. In the bottom panel, platelet dense granule release is indicated by ATP secretion. Lytic granule release from lymphocytes from P1 (**D**) was measured by determining the increase in the percentage of CD107a positive cytotoxic T-lymphocytes CTL (top panel) and natural killer (NK) cells (bottom panel) after stimulation with phytohaemagglutinin and anti-CD3 respectively. Data are also presented from 39 healthy controls with the median control value indicated by the horizontal line. Expression of AP-3 β3A was determined in P1 by immunoblotting protein extract from EBV-immortalised B-lymphoblastoid cells using an anti-β3A subunit antibody (**E**). Control data are presented from a healthy control (HC) and from an unrelated individual with genetically confirmed HPS2 (HPS2). Control experiments were performed using an anti-GAPDH antibody. Migration of relevant molecular mass protein markers are indicated on each immunoblot.

The index case is a female child of Lebanese origin (P1) with healthy consanguineous parents and two healthy older siblings (Figure 1A). She displayed reduced skin, hair and iris pigmentation compared to parents and siblings but was not dysmorphic (Figure 1B). Her clinical course has been dominated by recurrent severe febrile illnesses, including episodes of documented adenovirus and respiratory syncytial virus infection, on one occasion requiring ventilation on paediatric intensive care. At presentation, the index case was noted to have hepatosplenomegaly and fever, but had no other features of haemophagocytic syndrome (normal serum triglyceride and ferritin concentration and no morphological evidence of haemophagocytosis on a bone marrow aspirate). A high-resolution CT chest scan at 34 months old demonstrated patchy ground glass shadowing and interlobular septal thickening in the lower lobes suggestive of early stage pulmonary fibrosis. After follow up to 36 months, there have been no reported symptoms of abnormal bleeding or colitis.

Laboratory investigations revealed that P1 had persistent neutropenia (typically 0.2 × 10^9^/l; reference interval 1.5-7.0 × 10^9^/l), with normal neutrophil morphology in peripheral blood. Serum concentrations of immunoglobulin subclasses were normal and there were satisfactory antibody responses to childhood immunisations for haemophilus, tetanus and the 7-valent pneumococcal antigen suggesting normal B-lymphocyte function. Platelet impedance aggregation and secretion responses were monitored simultaneously using a CHRONO-LOG model 700 lumiaggregometer. Platelets from P1 displayed partial aggregation to 5 μg/ml collagen (maximum aggregation (MA) MA 14 Ω) and to 10 μM ADP (MA 9 Ω) whereas these agonists induced full aggregation of control platelets (MA 21 Ω and 24 Ω with 5 μg/ml collagen 10 μM ADP respectively; Figure 1C). There was brisk ATP secretion from control platelets in response to 5 μg/ml collagen and to10 μM ADP (2.34 nmol and 1.06 nmol ATP respectively). However, platelet ATP secretion was absent in P1 when stimulated with these agonists, indicating defective platelet dense granule release (Figure 1C).

A FACS-based granule release assay (GRA), using the surface expression of CD107a (lysosome-associated membrane protein 1 (LAMP-1)) as a marker of lytic granule exocytosis, was used to assess CTL and NK cells in a resting state, and after stimulation with anti-CD3 or phytohaemagglutinin respectively [14,15]. Granule secretion was determined by measuring the increase in the percentage of CD107a positive cells, determined from 100,000 events, in the resting state versus activated state. The percentage of CD107a positive NK cells in the resting state was higher in P1 (5.4%) compared to a healthy control (HC) measured in parallel (0.9%; Additional file 1: Figure S1). After cell stimulation, the proportion of CD107a positive CTL and NK cells from P1 increased by 0.8% and 5.5% respectively. These responses were within the lowest quartile of the 95% reference interval of responses determined at our laboratory by testing 39 healthy controls (Figure 1D).

As this clinical and laboratory phenotype was consistent with HPS2, immunoblots were performed to determine the expression level of the AP3 β3A-subunit in EBV-immortalised B lymphoblastoid cells from P1 (Prepared by the Health Protection Agency Culture Collections, Salisbury, UK). Cell lysates were resolved by electrophoresis on a sodium dodecyl sulphate-polyacrylamide gel (SDS-PAGE) and were immunoblotted with a rabbit polyclonal anti-β3A antibody (a gift from Professor Margaret Robinson, University of Cambridge) and a control rabbit polyclonal anti-GAPDH (Santa Cruz). In positive control cell lysates prepared from fresh healthy donor lymphocytes, immunoblotting demonstrated a single protein band of approximate molecular weight 123 kDa that is the expected molecular weight of the AP3 β3A-subunit. In contrast, there was no detectible full length AP3 β3A-subunit in lymphoblastoid cells from P1, or in a negative control lymphoblastoid cell lysates obtained from a previously reported patient with HPS2 [12] (Figure 1E).

**Figure 2:**
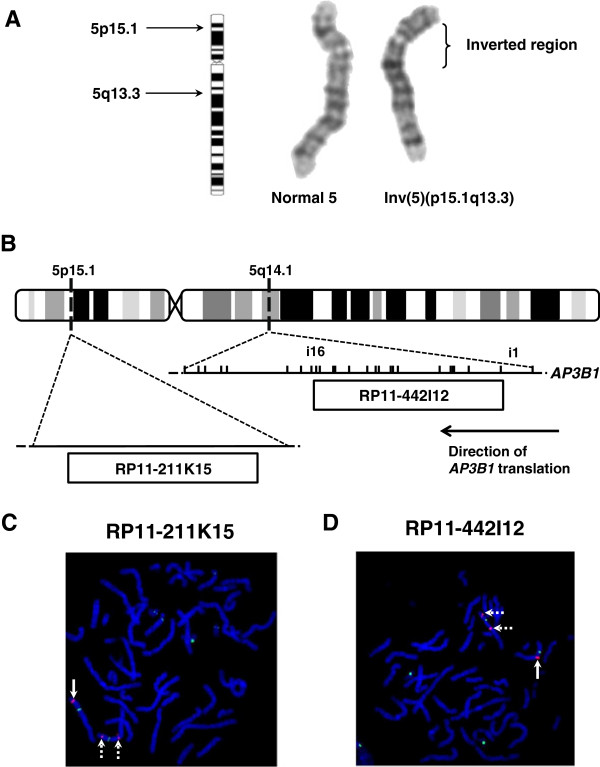
**Pericentric inversion of chromosome 5.** Partial ideogram of a metaphase from the bone marrow aspirate from P2 showing both the normal and abnormal Chr5 with an inversion estimated to be between cytobands 5p15.1 and 5q13.3 (**A**). Schematic diagram of Chr5 showing the normal cytoband localization of the fluorescence *in situ* hybridisation (FISH) probes RP11-211K15 and RP11-422I12 (**B**). RP11-211K15 maps to an intergenic region at 5p15.1. RP11-422I12 maps to 5q14.1, and spans intron 1 to intron 16 of *AP3B1*. FISH was performed on metaphase chromosomes from P2, who was a heterozygote carrier of the inverted Chr5 (**C** and **D**). The images show DAPI stained chromosomes labelled with pGA-16 (green signal) which is a centromere marker of Chr5 and Chr9. The 5p15.1 probe RP11-211K15 (**C**; red signal) correctly localises to 5p15.1 in the normal Ch5 (solid arrow) but showed dual hybridisation to 5q14.1 and 5p15.1 in the inverted Chr5 (broken arrows). The 5q14.1 probe RP11-422I12 (**D**; red signal) correctly localises to 5q14.1 in the normal Ch5 (solid arrow) but showed dual hybridisation to 5p15.1 and 5q14.1 in the inverted Chr5 (broken arrows).

Karyotype analysis of peripheral blood leukocyte metaphases from P1 demonstrated a pericentric inversion of Chr5, initially estimated to be inv(5)(p15.1q13.3), that was present in all observed Chr5, indicating a homozygous defect. An identical inversion of Chr5 was identified in heterozygous form in the asymptomatic mother of the index case (P2; Figure 1A and 2A). Microarray comparative genome hybridisation (cGH) using the Agilent OGT ISCA (International Standards for Cytogenomic Arrays) Consortium 8 × 60K version 2.0 probe set showed no loss of genetic material at 60 kb resolution, suggesting that the inversion was balanced (Additional file 2: Figure S2).

*AP3B1,* which encodes the AP-3 β3A-subunit, lies at cytoband 5q14.1 in the region of the initially estimated 5q inversion breakpoint. Therefore, we used fluorescence *in-situ* hybridisation (FISH) using probes prepared from the Sanger 30 kb EnsEMBL clone set to fine map both inversion breakpoints in metaphases prepared from P2, who displayed both normal and inverted Chr5. The hybridisation signal from the probe RP11-317N14 (NCBI 36 nomenclature), which maps telomeric from *AP3B1* intron (i) 6 to include exons (e)1-6 and the 5’ flanking sequence correctly localised to q14.1 of the inverted Chr5. However, hybridisation signals from probe RP11-59M8 (*AP3B1* i7 to i22), and probe RP11-51E14 (centromeric from *AP3B1* i17 to include e18-e26 and the 3^′^- flanking sequence) localized to 5p similar to the inv(5)(p15.1q13.3) observed in the initial diagnostic karyotype. Together, these observations suggested that the 5q breakpoint lay within *AP3B1*. This was confirmed by the detection of hybridisation signal from RP11-422I12 (*AP3B1* i1 to i16 (Chr5:77417815–77569358)) at both q14.1 and p15.1 of the inverted Chr5 but only at 5q in the normal Chr5 (Figure 2B and 2D).

The 5p inversion breakpoint was mapped using a similar strategy. In this analysis, the probe RP11-211K15, which maps to Chr5:18263045–18430126 within 5p15.1 displayed dual hybridisation to both the cytobands 5p15.1 and 15q14.1, consistent with the karyotype findings. RP11-211K15 maps to a genomic region on 5p that contains no identifiable genes in the RefSeq, UCSC or GENCODE databases (Figure 2B and 2C).

## Conclusions

We describe a new index case with reduced pigmentation and a platelet dense granule defect indicating an HPS group disorder. In addition, there was a clinical immunodeficiency phenotype, a persistent neutropenia and radiographic evidence suggestive of early pulmonary fibrosis. This phenotype was indicative of HPS2, which we confirmed by showing absent expression of AP-3 β3A subunit in lymphocytes by immunoblotting. It is noteworthy that the index case also displayed increased surface expression of lytic granule markers in resting CTL and NK cells and diminished granule release consistent with mis-trafficking of lytic granules. This is similar to, but less marked than in other secretopathies such as Chediak Higashi syndrome (OMIM #214500), Griscelli syndrome (OMIM #607624), and Familial haemophagocytic lymphohistiocytosis disorders (OMIM #608898 and #613101), which may be complicated by episodes of haemophagocytic syndrome in response to viral infection [16-18]. This is in keeping with other clinical descriptions of HPS2 in which haemophagocytic syndrome has been reported in only a minority of cases [7,8,12].

To date, the molecular pathogenesis of HPS2 has been reported in only 13 index cases. In these individuals, a total of 14 nucleotide variations within *AP3B1* have been identified, in all cases occurring as homozygous or compound heterozygous traits. These include 5 short deletion variations [10,12,19]; 3 nonsense single nucleotide variations (SNV) [7,9]; 2 insertions [8,20]; 2 splice site SNV [20,21]; 1 missense SNV [11] and 1 complex deletion-insertion [8], all of which are predicted to prevent expression of the AP-3 β3A subunit. Our finding that a previously unreported homozygous pericentric inversion (inv(5)(p15.1q14.1) was also associated with the HPS2 phenotype extends the repertoire of genetic variations associated with this disorder. We also demonstrated that the FISH probe RP11-422I12 hybridised to both the cytobands 5q14.1 and 5p15.1 thus localising the inversion breakpoint to within the boundaries of this probe. Since RP11-422I12 maps to *AP3B1* intron 1 to intron 16, the inversion necessarily lies within *AP3B1* and is predicted to disrupt gene expression. We cannot rule out the possibility of loss of genetic material within these boundaries in *AP3B1* related to the inversion. However, the array cGH data indicate that if any deletion occurred it is likely to be at less than 60kb size. Since we also mapped the 5p inversion breakpoint to a region of 5p15.1 that contained no CCDS-annotated genes, it is likely that the phenotype of the index case arose only from disruption of *AP3B1* at the 5q inversion breakpoint. This represents a novel molecular mechanism for HPS2.

## Consent

Written informed consent was obtained from the mother of the index case for publication of this case report and any accompanying images. A copy of the written consent is available for review by the series editor of this journal. Laboratory investigations were performed after approval from the UK NHS National Research Ethics Service (07/Q0704/48).

## Competing interests

The authors declared that they have no competing interests.

## Authors’ contributions

MLJ carried out cell culture and immunoblotting and co-wrote manuscript. SLM carried out genomic DNA extraction and cell culture. CB and VM carried out cytogenetics. RAM carried out platelet lumiaggregometry. KCG carried out granule release assays. VB, JF and SC collected clinical and laboratory phenotype data. AM directed the study, interpreted data and co-wrote manuscript. All authors read and approved the final manuscript.

## Pre-publication history

The pre-publication history for this paper can be accessed here:

http://www.biomedcentral.com/1471-2350/14/42/prepub

## Supplementary Material

Additional file 1: Figure S1 Granule release assay (GRA) results are shown for a normal healthy control (HC) and the index case (P1) for cytotoxic T-cells (CTLs) (A) and natural killer (NK) cells (B). Peripheral blood mononuclear cells were stimulated overnight with interleukin 2 followed by incubation with fluorescently-labelled anti-CD107a (LAMP-1) alone (resting; left panel A & B) or with anti-CD3 antibody to activate CTLs (right panel A) or phytohaemagglutinin (PHA) to activate NK cells (right panel B). Samples were analysed by flow cytometry, gating on lymphocytes by forward/side scatter. CD107a expression was analysed on CTLs and NK cells and the %CD107a + cells are indicated in each histogram. The increase in percentage of CD107a + cells (Δ %CD107a+) between resting and stimulated cells was determined. Click here for file

Additional file 2: Figure S2 Microarray comparative genome hybridisation profile of Chr5 from P1 (A). The expected position of signal from any loss of genetic material in the profile is indicated by (-) and potential gain of material is indicated by (+). A Chr 5 ideogram (B) is shown for comparison. Click here for file
