# Supplementary material for: Disruption of AP3B1 by a chromosome 5 inversion: a new disease mechanism in Hermansky-Pudlak syndrome type 2
Source: BMC Med Genet. 2013 Apr 4;14:42. doi: 10.1186/1471-2350-14-42 (PMC3663694; doi:10.1186/1471-2350-14-42)
Supplement: Additional file 1: Figure S1 — Granule release assay (GRA) results are shown for a normal healthy control (HC) and the index case (P1) for cytotoxic T-cells (CTLs) (A) and natural killer (NK) cells (B). Peripheral blood mononuclear cells were stimulated overnight with interleukin 2 followed by incubation with fluorescently-labelled anti-CD107a (LAMP-1) alone (resting; left panel A & B) or with anti-CD3 antibody to activate CTLs (right panel A) or phytohaemagglutinin (PHA) to activate NK cells (right panel B). Samples were analysed by flow cytometry, gating on lymphocytes by forward/side scatter. CD107a expression was analysed on CTLs and NK cells and the %CD107a + cells are indicated in each histogram. The increase in percentage of CD107a + cells (Δ %CD107a+) between resting and stimulated cells was determined. [file 1471-2350-14-42-S1.pptx]

## Slide 1
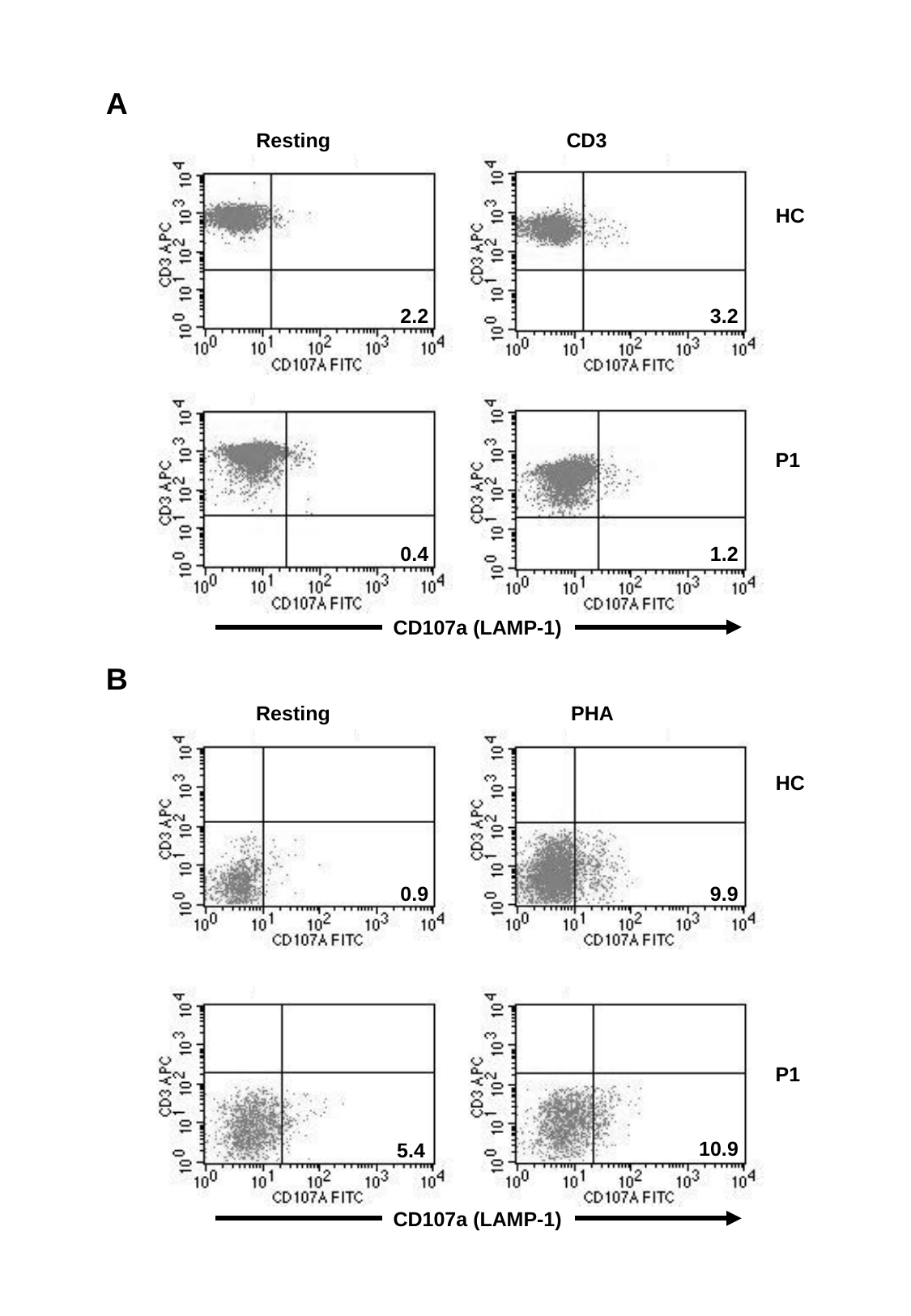

A
Resting
CD3
HC
P1
CD107a (LAMP-1)
B
Resting
PHA
HC
P1
CD107a (LAMP-1)
2.2
3.2
0.4
1.2
0.9
9.9
10.9
5.4

## Slide 2
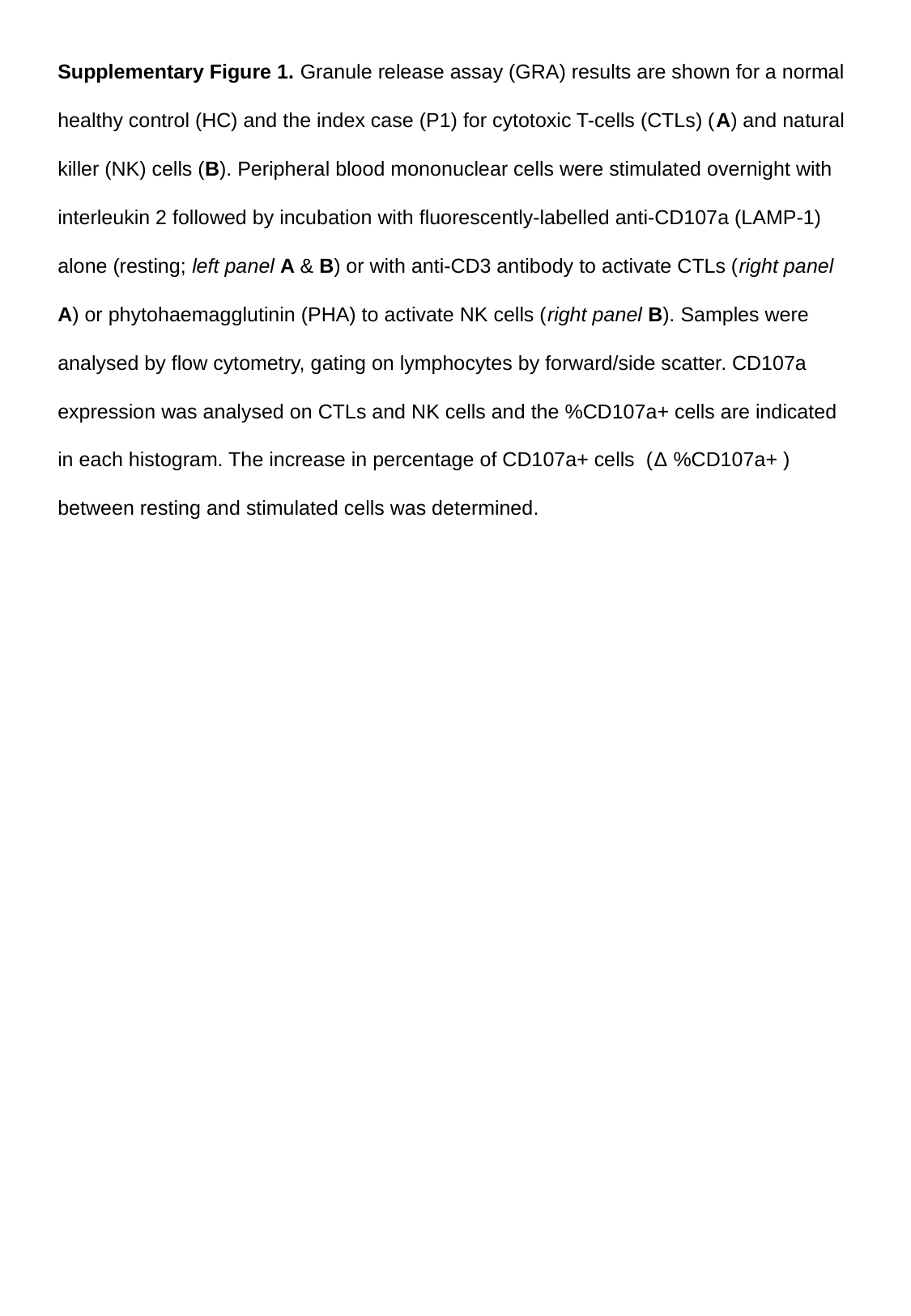

# Supplementary Figure 1. Granule release assay (GRA) results are shown for a normal healthy control (HC) and the index case (P1) for cytotoxic T-cells (CTLs) (A) and natural killer (NK) cells (B). Peripheral blood mononuclear cells were stimulated overnight with interleukin 2 followed by incubation with fluorescently-labelled anti-CD107a (LAMP-1) alone (resting; left panel A & B) or with anti-CD3 antibody to activate CTLs (right panel A) or phytohaemagglutinin (PHA) to activate NK cells (right panel B). Samples were analysed by flow cytometry, gating on lymphocytes by forward/side scatter. CD107a expression was analysed on CTLs and NK cells and the %CD107a+ cells are indicated in each histogram. The increase in percentage of CD107a+ cells (Δ %CD107a+ ) between resting and stimulated cells was determined.
