# Supplementary material for: Disruption of AP3B1 by a chromosome 5 inversion: a new disease mechanism in Hermansky-Pudlak syndrome type 2
Source: BMC Med Genet. 2013 Apr 4;14:42. doi: 10.1186/1471-2350-14-42 (PMC3663694; doi:10.1186/1471-2350-14-42)
Supplement: Additional file 2: Figure S2 — Microarray comparative genome hybridisation profile of Chr5 from P1 (A). The expected position of signal from any loss of genetic material in the profile is indicated by (-) and potential gain of material is indicated by (+). A Chr 5 ideogram (B) is shown for comparison. [file 1471-2350-14-42-S2.pptx]

## Slide 1
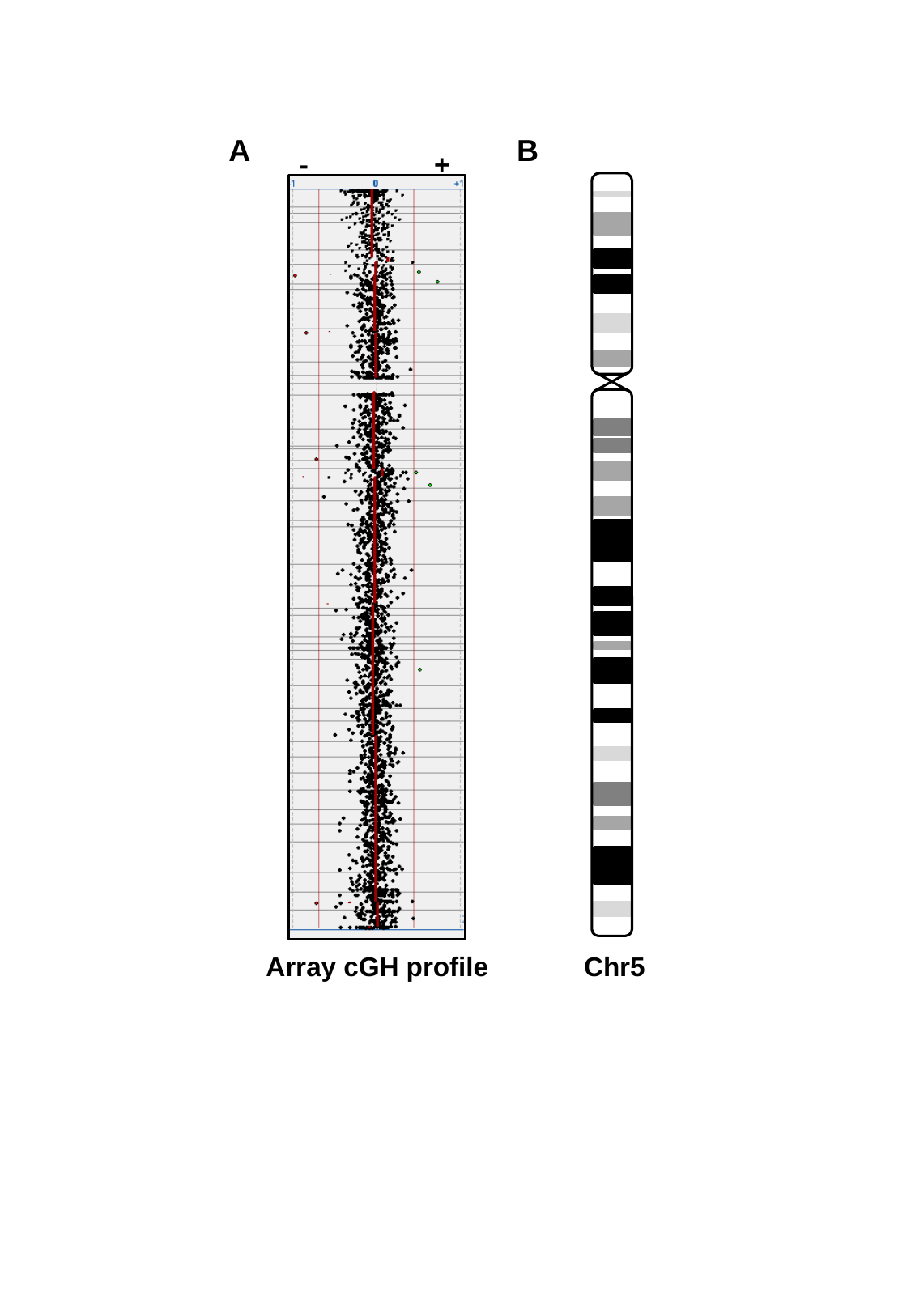

A
B
-
+
Chr5
Array cGH profile

## Slide 2
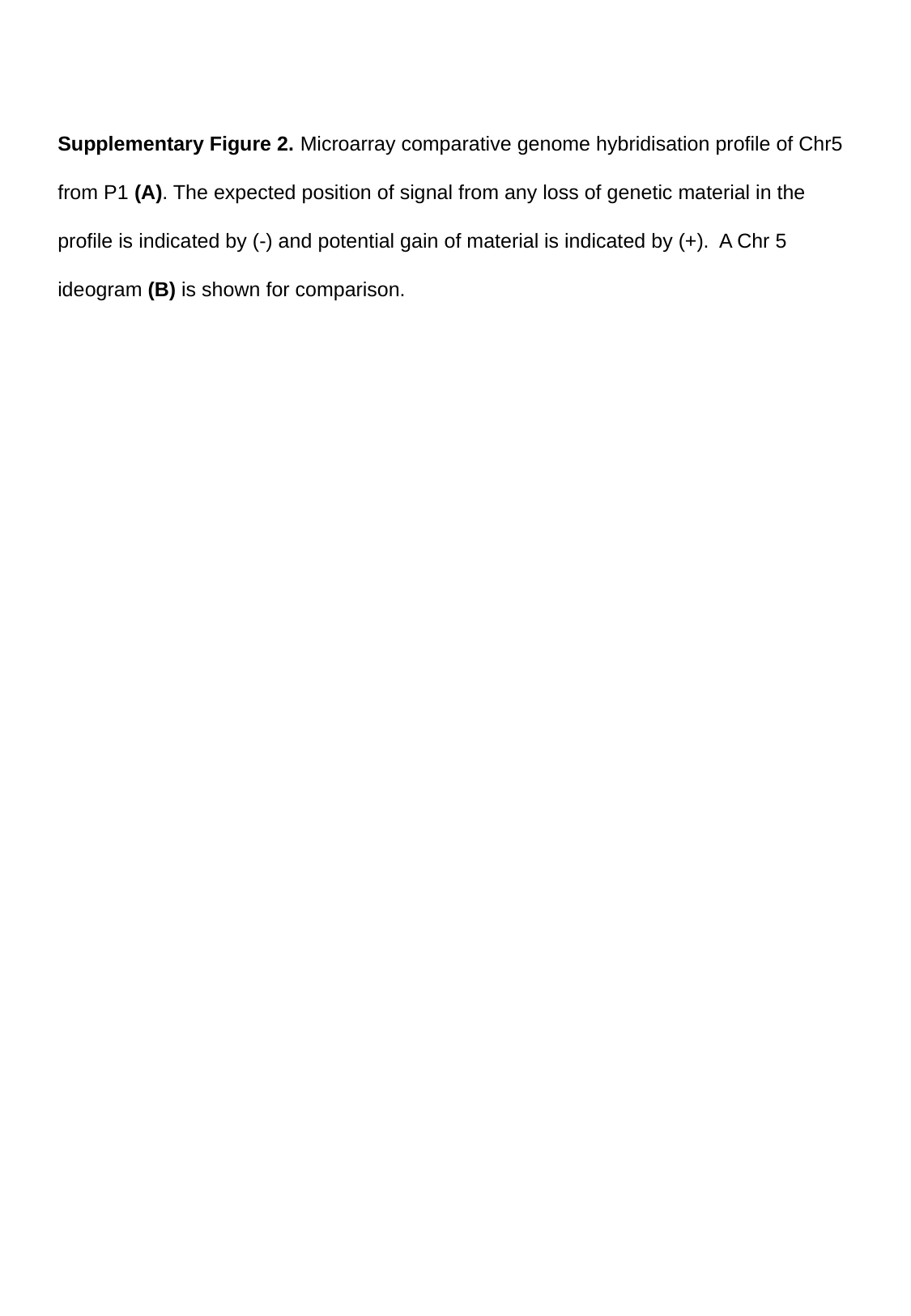

# Supplementary Figure 2. Microarray comparative genome hybridisation profile of Chr5 from P1 (A). The expected position of signal from any loss of genetic material in the profile is indicated by (-) and potential gain of material is indicated by (+). A Chr 5 ideogram (B) is shown for comparison.
